# Impact of smokeless tobacco packaging on perceptions and beliefs among youth, young adults, and adults in the U.S: findings from an internet-based cross-sectional survey

**DOI:** 10.1186/1477-7517-11-2

**Published:** 2014-01-17

**Authors:** Sarah E Adkison, Maansi Bansal-Travers, Danielle M Smith, Richard J O’Connor, Andrew J Hyland

**Affiliations:** 1 Roswell Park Cancer Institute, Department of Health Behavior, Elm & Carlton Streets Buffalo, New York 14263, USA

**Keywords:** Smokeless tobacco, Tobacco packaging, Health warning labels, Tobacco marketing

## Abstract

**Background:**

Research demonstrates that tobacco packaging elements (including health warning labels, descriptive characteristics, and corporate branding) are associated with knowledge of health risks and product appeal with cigarettes. Yet, little research has assessed this with smokeless tobacco (SLT) packaging. This study evaluates the association between three SLT packaging elements with knowledge of health risks and perceptions of novelty and appeal. Additionally, we assess how effects of these messages may differ across age groups, including youth (14-17 years), young adults (18-25 years), and older adults (26-65 years).

**Methods:**

1000 participants were administered a web-based survey in 2010 and shown three sets of SLT packs in random order, varied by descriptor (flavor descriptor vs. none), warning label format (graphic vs. text), and corporate branding (branded vs. plain packaging). Participants rated the packs compared with “no difference” on appeal, novelty, and risk perceptions associated with product use. Chi-square tests were used to test for significant differences in pack selections. Multinomial regression was employed to evaluate the association between effects of packaging elements and participant age.

**Results:**

More respondents selected the pack with the graphic warning label as the pack to make them consider the health risks associated with SLT use, attract their attention, and be least attractive to a smoker. The product with the text warning label was the product someone their age would want to be seen using and would appeal to peers. The SLT pack with the flavor descriptor was not associated with health risks associated with product use. The pack with corporate branding was selected as more appealing, to attract attention, and one they would want to be seen using; the plain pack was less attractive to smokers. Youth and young adults were more likely to indicate that pack elements affected their perceptions of appeal and risk associated with SLT products.

**Conclusion:**

These results suggest that SLT pack characteristics have a measurable effect on perceptions of health risk and product appeal. Future research should assess these findings in the context of harm reduction. Specifically, research is needed to determine whether pack elements on SLT products can effectively convey risk and harm.

## Background

Cigarette smoking and exposure to secondhand smoke remains the leading cause of preventable death in the United States killing 443,000 people in the U.S. annually [1,2]. Some research suggests that use of smokeless tobacco (SLT) products, such as chewing tobacco and snus, are less harmful and may serve as a potential cessation or substitution strategy for cigarette users [3,4]. However, while SLT products may pose less harm than conventional cigarettes, they are associated with increased risk for illnesses including oral cancer, esophageal cancer, pancreatic cancer, and heart disease, among others [5-8].

Due to the fact that SLT products do pose some level of harm, it is critical that advertising and marketing materials effectively convey this information to consumers. Given increased commercial marketing restrictions, product packaging has become one of the primary mechanisms for communications by the tobacco industry. Previous research on cigarette packaging has shown that elements of the package influence consumers’ ideas about appeal, novelty, and health risks associated with use [9-21]. Elements of tobacco packaging that are critical to industry communications include descriptive characteristics (e.g., strength, flavors) and corporate branding (name, colors, and structural design). Only one study to date has done so with SLT products [22].

### Warning labels

Health warning labels located on tobacco packages are one of the few and most cost-effective avenues available to governments and public health advocates to communicate the health risks associated with product use. Research on cigarette packaging consistently finds that highly visible health warnings labels are effective for informing consumers about the health risks associated with smoking and promoting cessation [10-12,23]. In addition, studies demonstrate that the influence of the public health warnings increase as the size of the warning labels increase [9], and pictorial health warnings are consistently more effective than text warnings alone [11,13,14]. To date, only one study has assessed how pictorial warning labels influence perceptions of SLT [22]. The findings from that study suggest that pictorial health warning labels are associated with reduced appeal of SLT products and increased perceived risks associated with use [22].

### Flavor descriptor terms

Research examining flavor descriptor terms on tobacco packaging shows that they have also been linked with product appeal and perceived health risks. In addition, tobacco flavors contribute to the palatability of tobacco products, making them more attractive to non-users and facilitating uptake [24]. The sale of flavored cigarettes, except menthol flavored cigarettes, was banned by the Family Smoking Prevention and Tobacco Control Act (FSPTCA) of 2009 because they are particularly appealing among youth. For example, research shows that 17-year-old smokers are three times more likely to use flavored cigarettes than smokers over age twenty-five [25].

Between 2000-2006, there was a 140% increase in the number of sub-brands of SLT from 20 in 2000 to 48 by 2006 [26], with the majority of these including some sort of flavor added to the tobacco product [27]. Furthermore, there is research that suggests use of menthol or mint flavorings in SLT products may be related to initiation and use of SLT [26]. Despite the rise in flavored SLT products, there remains a dearth of evidence about how flavors and their associated descriptor terms contribute to perceptions of appeal and risk associated with SLT product use.

### Corporate branding

Research suggests that plain packaging, which standardizes the appearance of tobacco products between brands and eliminates corporate imagery, may be an effective regulatory tool in diminishing brand appeal and reducing misperceptions about health risks that may be garnered from cigarette branding and package design [9,15,16]. For example, Wakefield and colleagues found that plain packaging was significantly more effective in reducing brand appeal than increasing the size of the public health warning, and that plain packaging lowered intentions to buy cigarettes [15]. Investigators have also demonstrated that plain packaging is effective in reducing false risk perceptions associated with branding and in reducing brand appeal, particularly among youth, for cigarettes [9,17]. Two systematic reviews of the literature (reviewing 54 studies that show the impact of plain tobacco packaging) have found that plain packaging as opposed to branded packs (1) reduces package appeal, (2) increases visibility and salience of health warnings, and (3) reduces confusion about the harm associated with use that has been shown to result from packs with corporate branding [28,29]. These findings prompted Australia to enact legislation in December 2012 that requires tobacco products be packaged in drab brown-colored “plain” packs, with the brand name and variety written in a standardized font. However, the research on plain packaging has not addressed smokeless tobacco. Additionally, the use of smokeless tobacco in Australia is virtually nonexistent and additional research is needed to assess the effects of plain packaging on perceptions and behaviors of SLT users [12].

Initiation of tobacco use most frequently occurs among youth – a group susceptible to messages and misleading information (e.g. tobacco package coloring or descriptors which may imply that a products is lower risk) presented on tobacco product packages [17,30,31]. Public health advocates have reiterated the importance of curbing youth uptake of tobacco, including combustible and non-combustible products, to avoid long and short-term health and social costs [32]. The importance of pack elements in communicating product information to consumers has been well documented in studies that focus on combustible tobacco products [9,11,15,33]. Evaluating the impact of packaging on perceptions of appeal and health risk among SLT products has been marked as a research need [34] given recent data showing increased rates of SLT use among youth and young adults. For example, the 2009 Youth Risk Behavior Surveillance reported that SLT use increased among high school students from 6.7% to 8.9% between 2003 and 2009, with an increase from 11% to 15% among male students (http://www.cdc.gov/mmwr/preview/mmwrhtml/ss5905a1.htm). Additionally, research shows that there was an increase from 13.6% to 15.4% among 18-25 year old non-Hispanic white men [35]. Increases in SLT use are particularly prominent among White high school males [32]. A primary focus of the FSPTCA is to discourage tobacco uptake and use among youth and young adults, and to regulate health warning labels on tobacco products to accurately convey the risks associated with use [27]. As it stands, some research suggests that young adults have little understanding of the risks associated with traditional and new SLT products [17,18,31]. More research is needed to evaluate how characteristics of SLT product packaging influence perceptions of health risks, particularly among high-risk groups like youth and young adults.

The current research evaluates the association between three SLT packaging elements –warning label format, flavor descriptors, and corporate branding – with perceptions of health risks, novelty and appeal. Additionally, because it is particularly important to curb tobacco uptake among youth and young adults, we assess how messages conveyed by these packaging elements may differ across age groups, including youth (14-17 years), young adults (18-25 years), and older adults (26-65 years).

## Methods

The study utilized a Web-based survey methodology and data were collected over a one-week period in July 2010. Participants were recruited from a panel maintained by Global Market Insite (http://www.gmi-mr.com/global-panel/index.php), a private company that maintains global consumer and specialty panels. Membership in their panel involves a double opt-in process where interested parties complete an online registration form, and then activate their account by clicking a link provided by GMI via e-mail. U.S. residents were targeted for inclusion. All participants were invited to respond to the survey via email and were deemed eligible if they were between the ages of 14 and 65 and provided consent. In the case of minors, parents were e-mailed a statement describing the survey risks and benefits of participation, compensation, and confidentiality prior to their child engaging in the survey. Parental consent and youth assent was obtained prior to participation in the survey. The sample was specifically designed to represent four age groups: 14-17 years (20%), 18-21 years (20%), 22-25 years (20%), and 25-65 years (40%). A total of 1000 participants responded to the survey. The study protocol was approved by the Institutional Review Board at Roswell Park Cancer Institute, Buffalo, NY.

### Procedure

**Figure 1:**
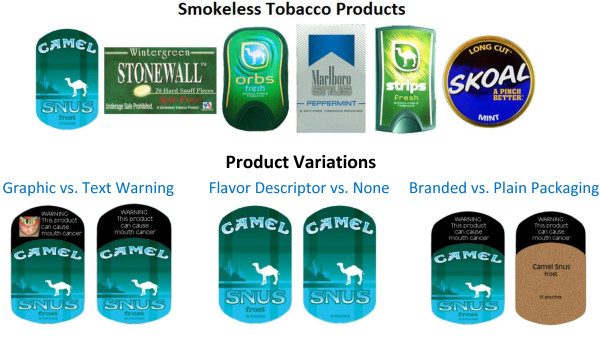
Smokeless tobacco products and product variations shown to participants.

Participants were first asked questions regarding their knowledge of the health risks associated with tobacco products and reasons for tobacco use. Following this series of questions, participants viewed, in random order, each of the products outlined in Figure 1. Six SLT products were included (Skoal Long Cut Mint, Camel Snus Frost, Marlboro Snus Peppermint, Camel Strips Fresh, Camel Orbs Fresh, and Stonewall Wintergreen Hard Snuff). Participants were provided with a brief one sentence description of how to use each product, given that many of the products may have been unfamiliar, and were then asked to indicate which product was the most appealing and which was the least appealing. The tobacco products selected as ‘most appealing’ and ‘least appealing’ were then presented to participants with three distinct packaging variations: graphic vs. text warning labels, flavor descriptor vs. no descriptor, and branded SLT pack vs. plain SLT pack (Figure 1). Participants were asked a series of questions regarding their perceptions of appeal and health risks of the displayed product based upon the different packaging formats (see Questions asked for each SLT packaging condition for the list of questions). The ordering of the packaging sets varied randomly between participants to minimize potential ordering effects. Because the most appealing product is one that participants would be more likely to use, the results reported focus on participants’ responses to the most appealing product selected. At the end of the session, participants were thanked and compensated with $5 in GMI "Market Points", which can be redeemed for a check mailed to them in USD.

Questions asked for each SLT packaging condition

All answer options were: Pack A, Pack B, No difference

Which pack would you expect to deliver the most dangerous chemicals?

Which pack would you expect to have the best taste?

Which pack do you think is the most likely to attract your attention?

Which pack do you think is the most dangerous to your health?

Which pack do you think would most appeal to people your age?

Which pack is most likely to make people think about the health risks of tobacco use?

Which pack do you think is least attractive to a smoker?

Which pack would someone your age most want to be seen using?

Which pack would you buy if you were trying to reduce health risks?

Which pack do you think contains smokeless tobacco of better quality (branded pack only)?

*Respondents were asked to pick between two packs or select ‘no difference’.

### Measures

#### ***Knowledge of health risks associated with smokeless tobacco***

Respondents were presented with a list of health effects and diseases that may or may not be caused by using smokeless tobacco. These health conditions included: lung cancer, oral cancer, pancreatic cancer, heart disease, emphysema, and lung disease. Respondents were asked, “Based on what you know or believe, does smokeless tobacco use cause…?” with a yes, no, or don’t know response option.

#### ***Package elements***

Upon selecting a product as most and least appealing, respondents were presented with each of three packaging conditions, individually tailored to reflect respondents’ selected most appealing product. Within each condition, respondents were presented with a series of questions and asked to select one pack within the condition for each question or indicate if there was no difference. Respondents were also presented with the three packaging conditions and asked this same series of questions for their selected least appealing product. Those results are not presented here, but largely mirrored the results for the most appealing product.

### Statistical analyses

Data were cleaned and analyzed using SPSS 21.0 (SPSS, Inc., Chicago, IL). Differences in demographic and tobacco use variables, as well as differences regarding knowledge of SLT and perceptions of appeal, novelty, and health risks associated with SLT pack design characteristics, were tested using chi-square tests of independence for each categorical variable. The chi-square test allows for comparisons among multiple groups. For example, in the case of branded packaging vs. plain packaging, respondents were given the option to select whether (1) the branded pack, (2) the plain pack, or (3) the packaging had ‘no difference’ on their opinion of the product. This test evaluates the hypothesis that the frequencies do not differ from their expected values (here specified to be equal across conditions such that each category would reflect 33.3% of respondents’ selections, χ^2^ statistic, *p-*value < 0.05). These analyses were performed for the overall sample and across age groups. We specifically assessed whether or not respondents endorsed a specific package design feature versus selecting “no difference” between packs to be associated with increased/decreased risk and appeal. Multinomial regression was employed to evaluate the association between packaging elements and participant age. These models were adjusted for sex and race/ethnicity (White non-Hispanic, Black non-Hispanic, Hispanic, other non-Hispanic), and tobacco use status. The outcome variable was the different pack selections (e.g. graphic, text, no difference [referent]). In other words, each of two variant options, (e.g. graphic and text warnings), were compared to selecting no difference between packs. The subgroups (e.g. age) were then compared on their likelihood of selecting a given option versus no difference between the two variants. While the data were specifically sampled to assess perceptions between different age groups, few significant differences were identified in knowledge related to SLT use and perceptions of risk and appeal between 18-21 year olds and 22-25 year olds. As a result, these two groups were collapsed into one to represent young adults for the analyses.

## Results

### Sample characteristics

Characteristics of the sample are presented in Table 1. The majority of the sample (74%) was White non-Hispanic, 7% were Black non-Hispanic, 11% were Hispanic, and 9% reported Other non-Hispanic. Half of the sample was female (50%). Three-fourths of respondents (75%) reported having tried a cigarette at some point in their lifetime, and half (50%) reported having smoked daily (at least one cigarette per day for 30 days). Nearly 17% of respondents reported using an SLT product within the past 30 days (used SLT at least 1 day within the past 30 days).

**Table 1:** Demographic characteristics (n = 1000)

**Demographic characteristic**	**Overall**^**1**^	**Used smokeless in past 30 days**^2^	**Smoked daily**^**2**^	**Intend to use smokeless in the next year**^2^
	**%**	**%**	**%**	**%**
**Overall**	100	16.5	49.6	13.3
**Age**				
14-17	20.1	11.9	14.9	17.9
18-25	39.9	24.3	56.9	37.6
26-65	40	11.0	59.8	18.8
χ^2^		**29.484**	**121.646**	**45.522**
**Gender**				
Female	50.1	12.4	53.5	17.0
Male	49.9	20.6	45.7	35.3
χ^2^		**12.398**	**6.087**	**43.428**
**Race/Ethnicity**				
White, non-Hispanic	74.1	12.8	51	23.1
Black, non-Hispanic	6.7	11.9	32.8	20.9
Hispanic	10.7	33.6	51.4	43.0
Other/non-Hispanic	8.5	30.6	48.2	35.3
χ^2^		**43.366**	**8.326**	**24.004**
**Ever smoked (even 2 puffs)**				
Yes	75.4	21.1	65.8	32.4
No	24.6	2.4	--	6.9
χ^2^		**46.819**		**62.288**
**Ever smoked daily**				
Yes	49.6	26	--	36.5
No	50.4	7.1	--	15.9
χ^2^		**64.575**		**55.101**
SLT use past 30 days^3^				
0 days	83.5	**--**	56.0	2.6
1-5 days	9.0	**--**	26.7	58.9
6-9 days	2.9	**--**	17.2	62.1
10-19 days	2.1	**--**	4.8	85.7
20-30 days	2.5	**--**	24.0	88.0
χ^2^			**68.156**	**520.876**

Bold indicates significant at p < .05; ^1^Data reflect column totals; ^2^Data reflect row totals, ^3^Respondents respondents were asked “During the past 30 days, on how many days did you use chewing tobacco, snuff, dip, or smokeless tobacco products?”.

Respondents who had used SLT in the past 30 days and intended to use SLT in the future were more likely to be between 18 and 25 years of age compared with other age groups, male, Hispanic, and report “Other” for their race. They were also more likely to have previously tried cigarette smoking and to have smoked daily.

Table 2 displays the SLT products that respondents selected as the most appealing overall and across the different age groups. Respondents’ overwhelmingly selected dissolvable tobacco (Camel Strips and Camel Orbs, 56%) as the most appealing products. Chi-square tests between age groups showed that young adults (18-25 year olds) were more likely to select Camel Strips (p = .033) and Camel Snus (p = 0.016) than older adults (26-65 year olds) and more likely to select Camel Snus (p = .039) than youth (14-17 year olds). Youth were more likely to select Stonewall (p = .048) than young adults. There were no significant differences in selections between youth and older adults. Older adults were more likely to select Marlboro Snus (p = .013) and Stonewall (p = .001) than young adults. Analyses also showed that the most appealing product selections varied based on tobacco use status for three products. Non-tobacco users were significantly more likely to select Camel Strips (p = .017) and Camel Orbs (p = .036) and tobacco users (use SLT or cigarettes) were more likely to select Camel Snus (p = .001) as the most appealing product (data not shown). In addition, men were more likely to select Stonewall (p = .024) and Skoal (p = .006) and women were more likely to select Camel Strips (p = .030).

**Table 2:** Most appealing products overall, by age, and by sex

	**Camel snus**	**Marlboro snus**	**Stonewall**	**Camel orbs**	**Camel strips**	**Skoal**
All	14.8%	13.4%	7.1%	23.4%	32.3%	9.0%
14-17 yrs.	11.4%	11.9%	7.5%	27.4%	34.3%	7.5%
18-25 yrs.	19.0%	10.5%	3.8%	21.3%	36.1%	9.3%
26-65 yrs.	12.3%	17.0%	10.3%	23.5%	27.5%	9.5%
Female	16.6%	11.6%	5.3%	24.3%	36.9%	5.3%
Male	16.5%	11.0%	5.0%	23.3%	35.5%	8.7%

### Knowledge of health effects associated with SLT use

The majority of respondents correctly identified that SLT use was associated with oral cancer (82%) and gum disease (82%). However, respondents were less likely to identify SLT use with heart disease (47%) and pancreatic cancer (30%). Approximately one-third incorrectly reported that SLT use was linked with emphysema (36%) and lung cancer (37%).

Common myths and other beliefs associated with tobacco use were also assessed (response options were: agree, neutral, disagree). Overall, 67% of respondents incorrectly reported that nicotine was a cause of cancer. This was highest among 14-17 year olds (77%) and lowest among 18-25 year olds (60%), while 26-65 year olds agreed 69% of the time. Nearly one-fifth (18.5%) believed that quitting smoking by 30 years of age eliminates the associated health risks, but no significant differences by age group were identified. Ten percent believed that so long as someone spits, SLT use is not dangerous; this finding was highest among 18-25 year olds (12.3%). Half (53%) believed that tobacco companies specifically target people their age, with youth (60%) and young adults (67%) significantly more likely to agree with this statement than 26-65 year olds (35%) (Youth χ^2^ (2, N = 601) = 35.832, p < .001); (Young adult χ^2^ (2, N = 799) = 91.934, p < .001).

### Packaging elements

#### ***Graphic vs. Text warning labels on SLT packaging***

**Figure 2:**
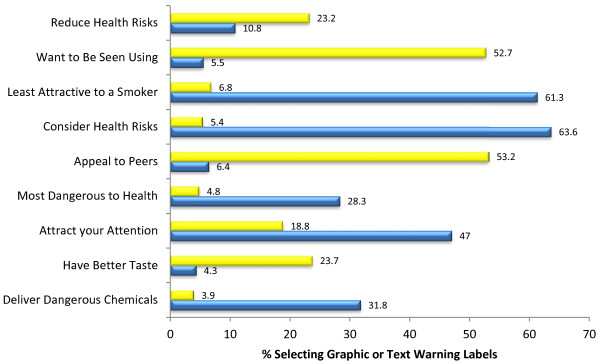
Perceptions of product health risk and appeal, based on warning label type (n = 1000), Yellow=Text, Blue=Graphic.

Figure 2 (and Table 3) displays participant responses for whether a SLT package with a graphic or text warning label was more likely to have an impact on measures of appeal and health risks associated with use. More respondents selected the pack with the graphic warning label as the pack to make them consider the health risks associated with SLT use, attract their attention, and be least attractive to a smoker. The product with the text warning label was selected as the product someone their age would want to be seen using and to appeal to peers.

**Table 3:** Perceptions of SLT product by pack conditions for the respondent’s most appealing product (%)

	**Condition**	**All**	**26-65**	**18-25**	**14-17**	**Condition**	**All**	**26-65**	**18-25**	**14-17**	**Condition**	**All**	**26-65**	**18-25**	**14-17**
Deliver dangerous chemicals	Graphic	31.8	27.3	36.8	30.8	Descriptor	7.3	6.5	10.0	3.5	Plain	25.3	19	30.8	26.9
	Text	3.9	2.8	5.0	4.0	None	10.8	7.3	13.5	12.4	Branded	5.0	3.2	7.0	4.5
	No difference	64.3	70.0	58.1	65.2	No difference	81.9	86.3	76.4	84.1	No difference	69.7	77.8	62.2	68.7
Have better taste	Graphic	4.3	4.3	5.3	2.5	Descriptor	46.5	37.3	53.9	50.2	Plain	3.7	3.0	5.3	2.0
	Text	23.7	17.5	29.6	24.4	None	6.6	7.8	7.5	2.5	Branded	52.5	41	62.4	55.7
	No difference	72.0	78.3	65.2	73.1	No difference	46.9	55.0	38.6	47	No difference	43.8	56	32.3	42.3
Attract your attention	Graphic	47.0	43.3	51.1	46.3	Descriptor	40.6	35.3	45.1	42.3	Plain	6.6	5	8.3	6.5
	Text	18.8	15.5	22.1	18.9	None	5.7	4.5	8.5	2.5	Branded	62.4	54.2	70.4	62.7
	No difference	34.2	41.3	26.8	34.8	No difference	53.7	60.3	46.4	55.2	No difference	31	40.8	21.3	30.8
Most dangerous to health	Graphic	28.3	23.3	32.6	19.9	Descriptor	5.6	5.0	7.5	3.0	Plain	20.8	14.2	25.3	24.9
	Text	4.8	5.0	5.3	3.5	None	10.5	7.0	14.0	10.4	Branded	7.3	4.2	11.5	5.0
	No difference	66.9	71.8	62.2	66.7	No difference	83.9	88	78.4	86.6	No difference	71.9	81.5	63.2	70.1
Appeal to peers	Graphic	6.4	5.5	7.5	6.0	Descriptor	38.8	27.8	46.6	45.3	Plain	3.9	2.2	5.3	4.5
	Text	53.2	42.8	63.2	54.2	None	5.2	5.5	6.8	1.5	Branded	61.7	50.8	72.9	61.2
	No difference	40.4	51.8	29.3	39.8	No difference	56.0	66.8	46.6	53.2	No difference	34.4	47	21.8	34.3
Consider health risks	Graphic	63.6	58.0	67.7	66.7	Descriptor	6.2	6.5	6.5	5.0	Plain	24.6	19.8	26.6	30.3
	Text	5.4	4.8	7.8	2.0	None	11.4	8.5	14.5	10.9	Branded	7.6	5.5	11.0	5.0
	No difference	31	37.3	24.6	31.3	No difference	82.4	85	78.9	84.1	No difference	67.8	74.8	62.4	64.7
Least attractive to smoker	Graphic	61.3	55.5	64.4	66.7	Descriptor	7.8	8.3	8.3	6.0	Plain	51.4	45.2	56.6	53.2
	Text	6.8	5.5	9.5	4.0	None	20.1	13.8	25.1	22.9	Branded	8.9	7.2	11.8	6.5
	No difference	31.9	39.0	26.1	29.4	No difference	72.1	78.0	66.7	71.1	No difference	39.7	47.5	31.6	40.3
Want to be seen using	Graphic	5.5	5.5	6.3	4.0	Descriptor	26.3	17.8	32.1	31.8	Plain	3.0	2.5	4.3	1.5
	Text	52.7	42.8	61.7	54.7	None	6.1	3.8	9.8	3.5	Branded	55.2	40.5	69.2	56.7
	No difference	41.8	51.8	32.1	41.3	No difference	67.6	78.5	58.1	64.7	No difference	41.8	57	26.6	41.8
Reduce health risks	Graphic	10.8	10.0	13.0	8.0	Descriptor	10.3	7.8	11.3	13.4	Plain	4.8	3.5	5.8	5.5
	Text	23.2	19.5	25.6	25.9	None	5.9	4.8	8.8	2.5	Branded	17.5	14.2	21.3	16.4
	No difference	66	70.5	61.4	66.2	No difference	83.8	87.5	79.9	84.1	No difference	77.7	82.2	72.9	78.1

Note: chi-square analyses demonstrated that all conditions were significant at p < .001.

Multinomial regression also revealed that graphic warning labels were particularly associated with conveying the health risks of tobacco use to youth and young adults as compared to older adults (26-65; see Additional file 1: Table S1), as compared to reporting no difference between types of warning. Compared to older adults, youth and young adults had greater odds of selecting the pack with the graphic warning label as dangerous to their health (OR: 1.521, CI: 1.026-2.555; OR: 1.434, CI: 1.033-1.992) and to make them consider the health risks associated with using the product (OR: 1.493, CI: 1.026-2.171; OR: 1.898, CI: 1.371-2.629), compared to reporting no difference between packs. Compared with older adults, youth and young adults noted the pack with the graphic as less attractive to smokers (OR: 1.738, CI: 1.191-2.537; OR: 1.782, CI: 1.293-2.456), while the one with the text warning was associated with increased odds of tasting better (OR: 1.698, CI: 1.110-2.599; OR: 1.280-2.584), appealing to peers (OR: 1.860, CI:1.293-2.675; OR: 2.669, CI: 1.951-3.653), and for someone to prefer to be seen using (OR: 1.783, CI: 1.242-2.552; OR: 2.326, CI: 1.707-3.169), again as compared to seeing no difference between warning types.

### Flavor descriptor

**Figure 3:**
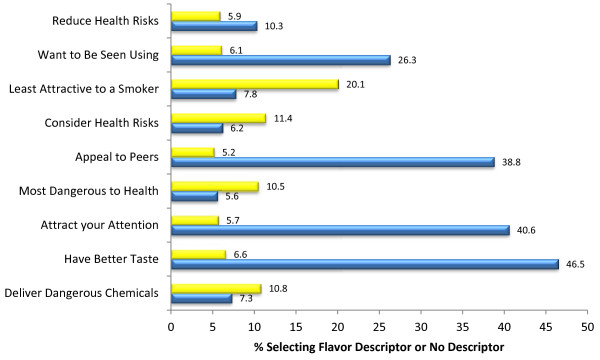
Perceptions of product health risk and appeal, based on presence of flavor descriptor (n = 1000), Yellow=Flavor descriptor, Blue=No Descriptor.

Figure 3 (and Table 3) displays the results of the impact of the flavor descriptor on perceptions of appeal and health risk associated with the SLT product that respondents selected as most appealing. The majority of respondents (over 50%) indicated that there was no difference between packaging elements on their product opinions regarding health risk and perceptions of appeal. Among those who selected a pack rather than “no difference”, the pack with the flavor descriptor was selected as having the best taste, to be mostly likely to attract their attention, and to be appealing to people their age.

Multinomial regression showed that youth, compared with older adults, were more likely to report the pack with the descriptor as having the best taste (OR: 1.695, CI: 1.185-2.423), that they want to be seen using (OR: 2.137, CI:1.427-3.199), that appeal to people their age (OR: 2.111, CI: 1.468-3.038), and reduce the health risks associated with use (OR: 1.762, CI: 1.005-3.087), compared to reporting no difference between packs (Additional file 2: Table S2). Young adults had increased odds of selecting the packaging with the flavor descriptor as attracting their attention (OR: 1.657, CI: 1.223-2.245), having the better taste (OR: 2.0065, CI: 1.519-2.806), to want to be seen using (OR: 2.355, CI: 1.1.664-3.333) and appealing to people their age (OR: 2.340, CI: 1.714-3.194) than older adults. Young adults also had increased odds of reporting the pack without the descriptor would deliver more dangerous chemicals (OR: 1.788, CI: 1.090-2.934) than older adults.

### Branded vs. Plain packaging

**Figure 4:**
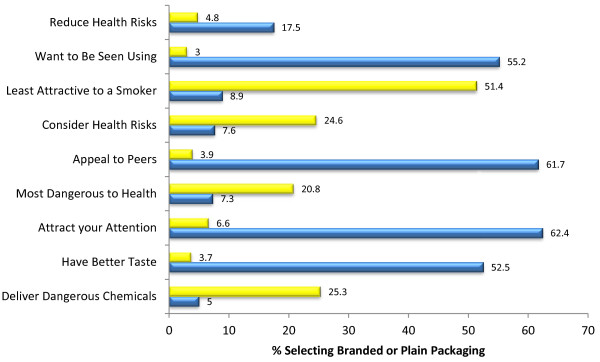
Perceptions of product health risk and appeal, based on presence of product branding (n = 1000), Yellow=Plain Packaging, Blue=Branded.

Figure 4 displays the results of the impact of the branded vs. plain packaging on perceptions of appeal and health risk of the SLT product respondents selected as most appealing. The pack that contained the corporate branding label was selected as having the best taste, more likely to attract respondents’ attention, more appealing to people their age, and the product that someone would want to be seen using. The plain packaging was selected as being less attractive to smokers. Additionally, the branded pack was reported to contain smokeless tobacco of better quality (χ^2^(N = 1000) = 388.142 expected = 333, observed = 401).

Multinomial regression showed that, compared to older adults, youth and young adults were more likely than older adults to select the plain pack, rather than saying there was ‘no difference between packs’, as having more dangerous chemicals (OR: 1.692, CI: 1.120-2.55; OR: 1.836, CI: 1.303-2.587, all report youth then young adults), being more dangerous to their health (OR: 2.039, CI: 1.312-3.168; OR: 2.126, CI: 1.459-3.098) to make them consider the health risks associated with use (OR:1.859, CI: 1.243-2.782; OR: 1.480, CI: 1.045-2.097) and less attractive to a smoker (OR: 1.451, CI: 1.012-2.080; 1.848, CI: 1.356-2.517). They selected the branded pack as having the best taste (OR: 1.960, CI: 1.374-2.797; OR: 2.558, CI: 1.887-3.468), more likely to attract their attention (OR: 1.646, CI: 1.132-2.394; OR: 2.503, CI: 1.800-3.482), and to want to be seen using (OR: 2.124, CI: 1.478-3.033; OR: 3.565, CI: 2.606-4.876). In general youth and young adults were more likely to select a pack as having an effect, rather than no difference, on their perceptions of harm and appeal in many of the areas assessed (see Additional file 3: Table S3).

## Discussion

Reducing tobacco-attributable illness in the U.S. and worldwide relies on effective tobacco control efforts, which includes adequately informing consumers about the dangers associated with tobacco use, especially vulnerable and susceptible youth. Research demonstrates that tobacco packaging elements (including health warning labels, descriptive characteristics, and corporate branding) are associated with knowledge of health risks and product appeal with cigarettes. Results from the current research suggest that package design characteristics are associated with perceptions of health risk and product appeal with smokeless tobacco packaging as well.

The current research found that graphic health warning labels were associated with lower ratings of product appeal and elicit greater concern for health risks than text warnings alone, consistent with previous research testing warning labels for cigarettes [11]. Furthermore, the impact of graphic warnings was strongest among the youth and young adults in our sample (14-25 year olds). Youth in our study reported that the products with graphic warnings would likely taste worse and pose more harm to a user, perceptions which may be linked with a lower likelihood of product use. A systematic review of literature on health warning labels conducted in 2011 indicated that health warning labels on cigarette packs can discourage youth uptake of tobacco, yet the impact of such warning labels is dependent upon the presence of imagery, location, size, and text of the warnings [36]. Research to date has focused on health warning labels on cigarette packs; the findings from this study suggest that this may also be true for warnings on SLT products.

Smokeless tobacco packaging with the flavor descriptor was not associated with conveying information regarding health risks associated with product use. This is consistent with previous findings with cigarettes – characterizing flavors do not necessarily alter risk perceptions around the product [37,38]. Future research should continue to evaluate how flavorings in smokeless tobacco products may be related to perceptions of health risks and appeal.

The branded pack was more appealing and more likely to grab respondents’ attention, while plain packaging was perceived as delivering more chemicals and making respondents consider the health risks associated with SLT use. Corporate branding on the packaging appeared to diminish perceptions of harm and increase positive perceptions of product quality compared to products in plain packaging. These findings are consistent with what other studies on combustible tobacco products have shown with regard to the influence of product packaging on consumer perceptions of the product [14,17,20,21,31]. In December 2012 plain packaging legislation was passed in Australia with the intent to reduce the appeal of packaging to consumers and increase the noticeability and salience of health warnings, among others (see Tobacco Plain Packaging Act 2011 for more detail: http://www.comlaw.gov.au/Details/C2011A00148). The real life effects of plain packaging on initiation, cessation, and relapse remain to be seen.

While this research illustrated that, among all age groups, SLT packaging elements were associated with product-related beliefs, a key finding was that youth and young adults were often more likely than older respondents to indicate that these elements would have an effect on their perceptions in each of the product conditions. This is of particular importance because, according to the most recent U.S. Surgeon General’s report, the majority of new smokeless tobacco users are youth and young adults [32]. If packaging elements are effective in conveying messages to young people, incorporating components that accurately convey the risk associated with use and reduce product appeal may result in a reduction in uptake among non-users.

Respondents overwhelmingly selected the new dissolvable tobacco products, Camel Strips and Camel Orbs, as the most appealing, accounting for over half of selections (62%). This product was only available in two test markets in the country at the time of this survey. Therefore, respondents found this product appealing simply by looking at the package and reading a one sentence description about how each product is used. Furthermore, when presented with the branded packaging condition, those who had selected the Strips and Orbs also selected the branded pack as particularly appealing to those their age and as the one they would want to be seen using. These tobacco products have packaging that closely resembles nontobacco products like breath strips or candy and may be alluring to youth. Further research should examine how these products are perceived after trial and how integrated corporate branding with other tobacco products (such as use of the Camel brand) influences perceptions and intention to try these products.

### Limitations

Several limitations should be considered. First, this was a web-based survey with an internet panel that was strategically designed to assess particular age groups, and does not reflect a representative sample of the US population. Because this survey was web-based, only those with access to a computer were able to participate, potentially underrepresenting individuals from lower socioeconomic classes. Additionally, because this was an opt-in internet panel, it is possible that there is some confounding present between internet access and panel composition.

Also, because this was a cross-sectional survey, we are unable to draw conclusions regarding the causation between packaging elements and perceptions of appeal and harm. The current data speak only to associations between packaging elements and perceptions. Another limitation is that tobacco use rates in this survey were markedly high and not reflective of the general population. Half of our sample had smoked daily at some point and three-quarters and smoked at some point in their lifetime. In addition, 17% of participants had used SLT in the past 30 days, though use in the general population among adults is 3.5%. Future research should apply these methods to a more broadly generalizable population. Despite these limitations, these findings highlight the importance of smokeless tobacco packaging in conveying information to consumers or creating impressions, and have important implications for future studies and tobacco control policy efforts.

## Conclusions

Smokeless tobacco packaging elements appear to be associated with perceptions of harm and product appeal, especially among young people. To date, only one other study has assessed how pictorial health warnings influence perceptions of smokeless tobacco with similar results [22], and, this is the first study to evaluate other elements of packaging including flavor descriptors and corporate branding. The findings are consistent with research on cigarettes showing that characteristics of package design convey information to consumers about the product [14,17,20,21,31]. Because this research demonstrated that packaging elements are particularly salient among youth and young adults, it highlights the importance of accurately conveying health risk information among those most susceptible to tobacco use uptake.

## Competing interests

Richard J. O’Connor (RJO) has served as a consultant to the Tobacco Constituents Subcommittee of the Tobacco Products Scientific Advisory Committee (TPSAC) of the U.S. Food and Drug Administration. RJO, via a subcontract from Research Triangle Institute, reviewed confidential and trade secret documents on menthol cigarettes submitted by tobacco manufacturers pursuant to an FDA request, and presented this information in closed session to TPSAC (10 Feb 2011); this information was not used in any way in the current study.

## Authors’ contributions

SA performed the statistical analysis, and wrote the first draft and revised the manuscript. MBT developed the study and its design, led data collection efforts, and contributed to the manuscript and interpretation of the data. RJO and DS contributed to the manuscript and provided assistance in interpretation of results. AH participated in the design of the study and revising the manuscript. All authors read and approved the final manuscript.

## Supplementary Material

Additional file 1: Table S1 Multinomial logistic regression for perceptions of SLT packaging with graphic or text health warning labels. Click here for file

Additional file 2: Table S2 Multinomial logistic regression for perceptions of SLT packaging with or without a flavor descriptor term. Click here for file

Additional file 3: Table S3 Multinomial logistic regression for perceptions of SLT packaging with or without corporate branding. Click here for file
