# Supplementary material for: Impact of smokeless tobacco packaging on perceptions and beliefs among youth, young adults, and adults in the U.S: findings from an internet-based cross-sectional survey
Source: Harm Reduct J. 2014 Jan 17;11:2. doi: 10.1186/1477-7517-11-2 (PMC3942180; doi:10.1186/1477-7517-11-2)
Supplement: Additional file 1: Table S1 — Multinomial logistic regression for perceptions of SLT packaging with graphic or text health warning labels. [file 1477-7517-11-2-S1.pdf]

Supplemental Table 1: Multinomial logistic regression for perceptions of SLT packaging with graphic or text health warning labels													
		**Deliver dangerous chemicals**				**Have the best taste**				**Attract attention**			
		**Graphic**		**Text**		**Graphic**		**Text**		**Graphic**		**Text**	
		OR	CI	OR	CI	OR	CI	OR	CI	OR	CI	OR	CI
Age	26-65	Ref		Ref		Ref		Ref		Ref		Ref	
	14-17	1.377	0.937-2.024	1.651	0.626-4.352	0.690	0.243-1.958	1.698*	1.110-2.599	1.356	0.922-1.994	1.671*	1.009-2.768
	18-25	1.464*	1.068-2.008	1.517	0.689-3.338	1.067	0.534-2.130	1.819***	1.280-2.584	1.872***	1.344-2.606	2.112***	1.383-3.225
Sex	Male	Ref		Ref		Ref		Ref		Ref		Ref	
	Female	0.832	0.634-1.093	1.314	0.676-2.558	1.042	0.557-1.946	0.858	0.636-1.156	1.098	0.828-1.455	0.801	0.557-1.150
Tobacco	Non-user	Ref		Ref		Ref		Ref		Ref		Ref	
	User	1.650***	1.231-2.211	2.086*	1.002-4.344	2.258*	1.127-4.525	1.677**	1.214-2.317	1.097	0.811-1.484	1.614*	1.093-2.382
Race	White	Ref		Ref		Ref		Ref		Ref		Ref	
	Other	0.868	0.519-1.452	4.850***	2.001-11.753	3.181**	1.325-7.636	0.905	0.522-1.569	0.776	0.469-1.284	0.514+	0.251-1.051
	Hispanic	1.047	0.670-1.637	3.818**	1.601-9.103	2.142+	0.847-5.248	0.980	0.605-1.587	0.652+	0.412-1.032	0.676	0.378-1.212
	Black	0.687	0.378-1.246	3.277*	1.140-9.416	3.169*	1.215-8.268	0.722	0.373-1.398	0.506*	0.287-0.892	0.685	0.343-1.370
		**Dangerous to health**				**Appeal to peers**				**Consider health risks**			
		**Graphic**		**Text**		**Graphic**		**Text**		**Graphic**		**Text**	
		OR	CI	OR	CI	OR	CI	OR	CI	OR	CI	OR	CI
Age	26-65	Ref		Ref		Ref		Ref		Ref		Ref	
	14-17	1.521*	1.026-2.555	0.801	0.322-1.993	1.814	0.829-3.969	1.860***	1.293-2.675	1.493*	1.026-2.171	0.463	0.147-1.455
	18-25	1.434*	1.033-1.992	0.851	0.435-1.661	1.760+	0.945-3.277	2.669***	1.951-3.653	1.898***	1.371-2.629	1.833+	0.944-3.559
Sex	Male	Ref		Ref		Ref		Ref		Ref		Ref	
	Female	0.849	0.641-1.125	1.155	0.636-2.099	2.089**	1.196-3.647	1.119	0.857-1.462	1.182	0.897-1.558	1.646	0.899-3.012
Tobacco	Non-user	Ref		Ref		Ref		Ref		Ref		Ref	
	User	1.569**	1.159-2.123	2.074*	1.079-3.986	3.139***	1.694-5.817	1.338*	1.006-1.780	1.097	0.817-1.473	1.625	0.848-3.115
Race	White	Ref		Ref		Ref		Ref		Ref		Ref	
	Other	1.085	0.648-1.818	3.731**	1.616-8.614	2.106+	0.959-4.629	0.562*	0.341-0.926	0.508**	0.308-0.838	3.385**	1.439-7.960
	Hispanic	1.164	0.737-1.839	3.471**	1.606-7.501	1.500	0.716*-3.143	0.514**	0.329-0.802	0.571*	0.360-0.905	5.200***	2.451-11.032
	Black	0.964	0.542-1.714	1.964	0.649-5.947	1.203	0.434-3.330	0.582*	0.341-0.991	0.470**	0.275-0.802	2.513+	0.914-6.910
		**Less attractive to smoker**				**Want to be seen using**				**Reduce health risks**			
		**Graphic**		**Text**		**Graphic**		**Text**		**Graphic**		**Text**	
		OR	CI	OR	CI	OR	CI	OR	CI	OR	CI	OR	CI
Age	26-65	Ref		Ref		Ref		Ref		Ref		Ref	
	14-17	1.738**	1.191-2.537	0.980	0.406-2.365	1.241	0.512-3.007	1.780**	1.242-2.552	0.932	0.493-1.763	1.553*	1.023-2.358
	18-25	1.782***	1.293-2.456	2.063*	1.124-3.784	1.201	0.626-2.306	2.326***	1.707-3.169	1.097	0.685-1.755	1.394+	0.979-1.986
Sex	Male	Ref		Ref		Ref		Ref		Ref		Ref	
	Female	1.219	0.927-1.605	1.048	0.615-1.786	1.358	0.762-2.421	1.448**	1.112-1.886	1.100	0.726-1.668	0.955	0.706-1.291
Tobacco	Non-user	Ref	Ref	Ref	Ref	Ref	Ref	Ref	Ref	Ref		Ref	
	User	1.212	0.904-1.625	1.566	0.882-2.780	4.135***	2.097-8.152	1.379*	1.404-1.830	2.201***	1.393-3.478	1.542**	1.114-2.135
Race	White	Ref		Ref		Ref		Ref		Ref		Ref	
	Other	0.550*	0.332-0.909	2.164*	1.012-4.626	2.904**	1.354-6.230	0.514**	0.311-0.850	2.461**	1.319-4.590	0.671	0.360-1.248
	Hispanic	0.634*	0.405-0.993	1.782	0.839-3.782	0.683	0.227-2.050	0.788	0.513-1.211	2.536***	1.432-4.492	0.936	0.560-1.562
	Black	0.643	0.371-1.114	2.127+	0.877-5.158	2.227+	0.889-5.576	0.641	0.374-1.098	2.185*	1.030-4.634	1.178	0.648-2.141
Note: “No Difference” is the referent; +p<.10, *p<.05, **p<.01, ***p<.001													
