# Supplementary material for: Impact of smokeless tobacco packaging on perceptions and beliefs among youth, young adults, and adults in the U.S: findings from an internet-based cross-sectional survey
Source: Harm Reduct J. 2014 Jan 17;11:2. doi: 10.1186/1477-7517-11-2 (PMC3942180; doi:10.1186/1477-7517-11-2)
Supplement: Additional file 2: Table S2 — Multinomial logistic regression for perceptions of SLT packaging with or without a flavor descriptor term. [file 1477-7517-11-2-S2.pdf]

Supplemental Table 2: Multinomial logistic regression for perceptions of SLT packaging with or without a flavor descriptor term

		Deliver dangerous chemicals				Have the best taste				Attract attention			
		Descriptor Term		None		Descriptor Term		None		Descriptor Term		None	
		OR	CI	OR	CI	OR	CI	OR	CI	OR	CI	OR	CI
Age	26-65	Ref		Ref		Ref		Ref		Ref		Ref	
	14-17	0.641	0.268-1.536	1.771+	0.989-3.171	1.695**	1.185-2.423	0.439	0.162-1.188	1.381+	0.965-1.976	0.698	0.246-1.981
	18-25	1.313	0.765-2.253	1.788*	1.090-2.934	2.065***	1.519-2.806	0.990	0.558-1.756	1.657***	1.223-2.245	1.739+	0.926-3.263
Sex	Male	Ref		Ref		Ref		Ref		Ref		Ref	
	Female	0.851	0.522-1.386	0.768	0.510-1.156	1.200	0.924-1.559	1.255	0.742-2.126	0.917	0.707-1.190	0.978	0.558-1.716
Tobacco	Non-user	Ref		Ref		Ref		Ref		Ref		Ref	
	User	2.452**	1.015-4.368	1.455+	0.934-2.268	1.292+	0.977-1.710	2.943***	1.617-5.358	1.120	0.848-1.480	2.908**	1.498-5.646
Race	White	Ref		Ref		Ref		Ref		Ref		Ref	
	Other	2.105*	1.015-4.368	1.47	0.728-2.968	0.522**	0.318-0.858	1.623	0.718-3.666	0.841	0.516-1.372	2.691*	1.193-6.072
	Hispanic	1.491	0.694-3.205	2.527***	1.463-4.366	0.862	0.554-1.341	2.504*	1.239-5.061	0.904	0.585-1.394	2.214*	1.004-4.882
	Black	2.469*	1.086-5.613	1.929+	0.926-4.018	0.738	0.435-1.253	1.398	0.508-3.848	0.552	0.309-0.985	3.466**	1.496-8.026
		Dangerous to health				Appeal to peers				Consider health risks			
		Descriptor Term		None		Descriptor Term		None		Descriptor Term		None	
	OR	CI	OR	CI	OR	CI	OR	CI	OR	CI	OR	CI	OR
Age	26-65	Ref		Ref		Ref		Ref		Ref		Ref	
	14-17	0.736	0.282-1.920	1.458	0.787-2.699	2.111***	1.468-3.038	0.333+	0.096-1.163	0.931	0.424-2.042	1.260	0.701-2.264
	18-25	1.277	0.695-2.348	1.833*	1.110-3.026	2.340***	1.714-3.194	1.307	0.698-2.445	0.688	0.378-1.253	1.512+	0.942-2.428
Sex	Male	Ref		Ref		Ref		Ref		Ref		Ref	
	Female	1.196	0.687-2.083	0.646*	0.424-0.987	0.946	0.727-1.232	0.889	0.496-1.592	1.267	0.743-2.161	0.606*	0.403-0.911
Tobacco	Non-user	Ref		Ref		Ref		Ref		Ref		Ref	
	User	3.080***	1.597-5.941	1.457	0.926-2.291	1.147	0.864-1.542	1.967*	1.031-3.750	3.204***	1.732-5.926	1.384	0.898-2.132
Race	White	Ref		Ref		Ref		Ref		Ref		Ref	
	Other	1.597	0.633-4.028	1.911+	0.977-3.738	0.935	0.577-1.515	1.945	0.742-5.096	4.613***	2.246-9.474	2.066*	1.075-3.970
	Hispanic	2.123+	0.965-4.669	3.442***	1.997-5.934	1.007	0.644-1.576	4.638***	2.265-9.500	2.870**	1.402-5.876	2.598***	1.490-4.529
	Black	3.725**	1.600-8.677	2.961**	1.469-5.967	0.948	0.552-1.629	3.049*	1.160-8.014	1.662	0.559-4.942	2.818**	1.463-5.429
		Less attractive to smoker				Want to be seen using				Reduce health risks			
		Descriptor Term		None		Descriptor Term		None		Descriptor Term		None	
		OR	CI	OR	CI	OR	CI	OR	CI	OR	CI	OR	CI
Age	26-65	Ref		Ref		Ref		Ref		Ref		Ref	
	14-17	0.763	0.375-1.553	1.771*	1.129-2.777	2.137***	1.427-3.199	1.184	0.456-3.078	1.762*	1.005-3.087	0.569	0.203-1.594
	18-25	0.931	0.543-1.594	1.968***	1.344-2.881	2.355***	1.664-3.333	2.554**	1.333-4.893	1.480	0.899-2.438	1.398	0.759-2.576
Sex	Male	Ref		Ref		Ref		Ref		Ref		Ref	
	Female	0.720	0.446-1.163	1.058	0.770-1.454	1.076	0.804-1.439	1.121	0.648-1.941	0.860	0.569-1.299	1.080	0.625-1.866
Tobacco	Non-user	Ref		Ref		Ref		Ref		Ref		Ref	
	User	1.398	0.842-2.323	1.133	0.803-1.597	1.090	0.797-1.490	2.375**	1.257-4.488	1.155	0.740-1.804	2.329**	1.243-4.363
Race	White	Ref		Ref		Ref		Ref		Ref		Ref	
	Other	2.535*	1.217-5.277	1.302	0.741-2.289	1.025	0.600-1.752	3.145**	1.401-7.062	1.226	0.579-2.596	4.242***	1.992-9.032
	Hispanic	3.175***	1.655-6.091	2.002**	1.241-3.231	1.588+	0.991-2.543	6.440***	3.281-12.638	1.546	0.827-2.891	4.161***	2.050-8.442
	Black	3.160**	1.429-6.989	2.001*	1.114-3.594	1.688+	0.969-2.942	4.260**	1.700-10.679	2.049*	1.012-4.150	4.687***	1.978-11.110

Note: “No Difference” is the referent; +p<.10, *p<.05, **p<.01, ***p<.001
