# Supplementary material for: Impact of smokeless tobacco packaging on perceptions and beliefs among youth, young adults, and adults in the U.S: findings from an internet-based cross-sectional survey
Source: Harm Reduct J. 2014 Jan 17;11:2. doi: 10.1186/1477-7517-11-2 (PMC3942180; doi:10.1186/1477-7517-11-2)
Supplement: Additional file 3: Table S3 — Multinomial logistic regression for perceptions of SLT packaging with or without corporate branding. [file 1477-7517-11-2-S3.pdf]

Supplemental Table 3: Multinomial logistic regression for perceptions of SLT packaging with or without corporate branding

		Deliver dangerous chemicals				Have the best taste				Attract your attention			
		Plain Pack		Branded Pack		Plain Pack		Branded Pack		Plain Pack		Branded Pack	
		OR	CI	OR	CI	OR	CI	OR	CI	OR	CI	OR	CI
Age	26-65	Ref		Ref		Ref		Ref		Ref		Ref	
	14-17	1.692*	1.120-2.556	1.683	0.680-4.167	0.897	0.273-2.950	1.960***	1.374-2.797	1.895	0.870-4.130	1.646**	1.132-2.394
	18-25	1.836***	1.303-2.587	1.981+	0.979-4.008	2.274*	1.051-4.923	2.558***	1.887-3.468	2.524**	1.334-4.774	2.503***	1.800-3.482
Sex	Male	Ref		Ref		Ref		Ref		Ref		Ref	
	Female	0.814	0.607-1.090	1.070	0.594-1.926	0.955	0.480-1.897	1.192	0.919-1.547	0.966	0.563-1.657	1.124	0.851-1.484
Tobacco	Non-user	Ref		Ref		Ref		Ref		Ref		Ref	
	User	1.421*	1.038-1.946	2.247*	1.153-4.378	2.052+	0.958-4.393	1.281+	0.970-1.692	1.997*	1.103-3.618	1.208	0.898-1.626
Race	White	Ref		Ref		Ref		Ref		Ref		Ref	
	Other	1.103	0.650-1.871	2.920*	1.222-6.975	2.121	0.718-6.266	0.892	0.550-1.446	1.338	0.553-3.238	0.694	0.416-1.157
	Hispanic	1.314	0.822-2.099	3.765***	1.768-8.016	2.606*	1.050-6.470	0.710	0.460-1.095	1.923+	0.932-3.966	0.676	0.427-1.072
	Black	1.468	0.830-2.597	4.200**	1.681-10.494	4.312**	1.636-11.37	0.700	0.408-1.199	1.388	0.527-3.660	0.668	0.386-1.156
		Dangerous to health				Appeal to peers				Consider health risks			
		Plain Pack		Branded Pack		Plain Pack		Branded Pack		Plain Pack		Branded Pack	
		OR	CI	OR	CI	OR	CI	OR	CI	OR	CI	OR	CI
Age	26-65	Ref	Ref	Ref	Ref	Ref	Ref	Ref	Ref	Ref	Ref	Ref	Ref
	14-17	2.039**	1.312-3.168	1.512	0.659-3.470	2.791*	1.039-7.498	1.691**	1.176-2.432	1.859**	1.243-2.782	1.199	0.537-2.677
	18-25	2.126***	1.459-3.098	2.533**	1.385-4.630	4.020***	1.712-9.441	3.234***	2.337-4.475	1.480*	1.045-2.097	1.698+	0.966-2.987
Sex	Male	Ref	Ref	Ref	Ref	Ref	Ref	Ref	Ref	Ref	Ref	Ref	Ref
	Female	0.827	0.603-1.133	0.761	0.461-1.255	0.623	0.311-1.250	1.164	0.887-1.527	0.869	0.647-1.167	0.851	0.522-1.390
Tobacco	Non-user	Ref	Ref	Ref	Ref	Ref	Ref	Ref	Ref	Ref	Ref	Ref	Ref
	User	1.292	0.920-1.815	2.327**	1.316-4.115	1.586	0.753-3.338	1.030	0.771-1.377	1.384*	1.007-1.903	2.614***	1.495-4.571
Race	White	Ref	Ref	Ref	Ref	Ref	Ref	Ref	Ref	Ref	Ref	Ref	Ref
	Other	1.006	0.553-1.829	2.998**	1.489-6.037	1.651	0.596-4.570	0.672	0.407-1.109	0.879	0.492-1.571	3.186***	1.611-6.302
	Hispanic	1.679*	1.033-2.729	0.311***	1.592-6.073	1.956	0.795-4.816	0.722	0.461-1.130	1.484+	0.939-2.346	2.046+	0.985-4.250
	Black	2.424**	1.383-4.249	3.016*	1.238-7.348	1.98	0.614-6.388	0.936	0.537-1.630	1.356	0.750-2.449	4.119***	1.923-8.825
		Less attractive to smoker				Want to be seen using				Reduce health risks			
		Plain Pack		Branded Pack		Plain Pack		Branded Pack		Plain Pack		Branded Pack	
		OR	CI	OR	CI	OR	CI	OR	CI	OR	CI	OR	CI
Age	26-65	Ref	Ref	Ref	Ref	Ref	Ref	Ref	Ref	Ref	Ref	Ref	Ref
	14-17	1.451*	1.012-2.080	1.059	0.514-2.184	0.752	0.198-2.860	2.124***	1.478-3.033	1.866	0.799-4.354	1.320	0.815-2.137
	18-25	1.848***	1.356-2.517	1.876*	1.093-3.220	2.987*	1.278-6.985	3.565***	2.606-4.876	1.212	0.592-2.481	1.425+	0.970-2.094
Sex	Male	Ref	Ref	Ref	Ref	Ref	Ref	Ref	Ref	Ref	Ref	Ref	Ref
	Female	1.061	0.814-1.382	0.690	0.429-1.110	1.297	0.610-2.759	1.153	0.884-1.504	0.658	0.359-1.207	1.021	0.732-1.423
Tobacco	Non-user	Ref	Ref	Ref	Ref	Ref	Ref	Ref	Ref	Ref	Ref	Ref	Ref
	User	1.180	0.889-1.567	1.636+	0.984-2.719	1.192	0.537-2.644	1.403*	1.057-1.862	2.717**	1.372-5.382	1.667**	1.163-2.389
Race	White	Ref	Ref	Ref	Ref	Ref	Ref	Ref	Ref	Ref	Ref	Ref	Ref
	Other	0.788	0.476-1.303	2.405*	1.186-4.878	2.395	0.791-7.249	0.729	0.445-1.194	3.779**	1.627-8.778	1.542	0.875-2.716
	Hispanic	0.912	0.581-1.429	2.480**	1.283-4.795	3.242*	1.265-8.314	0.659+	0.423-1.025	3.364**	1.556-7.270	1.371	0.814-2.310
	Black	0.873	0.505-1.510	2.460*	1.102-5.491	2.199	0.590-8.196	0.804	0.472-1.370	2.877*	1.034-8.005	1.783+	0.971-3.274

Note: “No Difference” is the referent; +p<.10, *p<.05, **p<.01, ***p<.001
